# Cathepsin-mediated regulation of alpha-synuclein in Parkinson’s disease: a Mendelian randomization study

**DOI:** 10.3389/fnagi.2024.1394807

**Published:** 2024-05-30

**Authors:** Liyu Lin, Zilun Wu, Haocheng Luo, Yunxuan Huang

**Affiliations:** ^1^The First Clinical Medical College of Guangzhou University of Chinese Medicine, Guangzhou, Guangdong, China; ^2^The First Affiliated Hospital of Guangzhou University of Chinese Medicine, Guangzhou, Guangdong, China

**Keywords:** Parkinson’s disease, cathepsin, alpha-synuclein, Mendelian randomization, meta-analysis

## Abstract

**Objective:**

The observational association between cathepsin and Parkinson’s disease (PD) has been partially explored in previous research. However, the causal relationship remains unclear. In this study, our objective is to investigate the causal link between cathepsin and PD using Mendelian randomization (MR) analysis and elucidate the underlying mechanisms governing their interaction.

**Methods:**

Utilizing bidirectional two-sample MR and multivariable MR, we systematically investigates the causal relationship between nine cathepsins and PD. The data pertaining to cathepsins were obtained from the Integrative Epidemiology Unit (IEU) Open GWAS Project, while data related to PD were sourced from versions R9 and R10 of the FinnGen database. The primary analytical method utilized was the inverse variance weighted (IVW), with MR analysis initially conducted using PD data from R9, complemented by a series of sensitivity analyses. Subsequently, replication analysis was performed on the R10 dataset, and meta-analysis were employed to merge the findings from both datasets. To explore potential mechanisms by which Cathepsins may impact PD, MR analyses were performed on significant Cathepsins with alpha-synuclein. MR analysis and colocalization analysis were conducted on expression quantitative trait loci (eQTL) data of gene related to alpha-synuclein with PD data.

**Result:**

Forward MR analyses revealed more cathepsin B (CTSB) associated with less PD risk (OR = 0.898, 95%CI: 0.834–0.966, *p* = 0.004), while more cathepsin H (CTSH) (OR = 1.076, 95%CI: 1.007–1.149, *p* = 0.029) and more cathepsin S (CTSS) (OR = 1.076, 95%CI: 1.007–1.150, *p* = 0.030) associated with increasing PD risk. Meta-analyses validated these associations. Multivariate MR Results were consistent with those before adjustment. No significant results were observed in bidirectional MR analysis. In the investigation of the underlying mechanism, our findings demonstrate that CTSB significantly reduces the levels of alpha-synuclein (OR = 0.909, 95%CI: 0.841–0.983, *p* = 0.017). Concurrently, a genetically determined positive correlation between alpha-synuclein and PD is illuminated by both eQTL MR and colocalization analysis.

**Conclusion:**

In conclusion, this MR study yields robust evidence suggesting an association between elevated levels of CTSB and reduced PD risk, mediated by the downregulation of alpha-synuclein levels. Conversely, higher levels of CTSH and CTSS are associated with an increased risk of PD. These findings offer novel insights into the pathophysiological mechanisms of PD and identify potential drug targets for disease prevention and treatment warranting further clinical investigations.

## 1 Introduction

Parkinson’s disease (PD) is a prevalent neurodegenerative disorder characterized by the loss of dopaminergic neurons in the substantia nigra region of the brain. As the second most common neurodegenerative disease, PD affects millions of people worldwide (Bloem et al., 2021). Despite extensive research, the precise etiology and pathogenic mechanisms of PD remain unclear. Current studies widely posit that aberrant aggregation of alpha-synuclein is a crucial factor in the onset of PD (Dehay et al., 2015). Emerging evidence suggests that proteases, specifically cathepsins, may play a crucial role in the development and progression of PD.

Cathepsins, a group of lysosomal proteases, play a critical role in the breakdown of proteins into smaller peptides or amino acids. These enzymes are pivotal in numerous physiological processes such as protein degradation, cell signaling, and the regulation of immune responses (Khaket et al., 2019). In recent years, Cathepsins have been identified beyond the confines of lysosomes, exhibiting widespread presence in the cell nucleus, mitochondria, cytoplasm, and extracellular spaces (Vizovišek et al., 2019; Wang et al., 2023). They are considered pivotal signaling molecules, playing crucial roles in the mechanisms underlying neurodegenerative disorders and cancer. Hence, there has been a growing focus on understanding the role of cathepsins in neurodegenerative diseases, including PD (Moors et al., 2016). Among these, Cathepsin B (CTSB) has been found to be closely associated with PD at multiple genetic levels. For instance, in a genetic sequencing study by Milanowski et al. (2022), an elevated mutation rate of the CTSB p.Gly284Val locus was observed in PD populations. Additionally, in a primary genome-wide association study (GWAS) by Blauwendraat et al. (2020), variants near CTSB were identified as one of the most significant contributors to PD occurrence.

Utilizing genetic variants as instrumental variables (IVs), Mendelian Randomization (MR) is an increasingly popular analytical technique employed to infer the causal impact of exposures on outcomes, providing insights into their potential causal effects (Smith and Ebrahim, 2003; Pierce et al., 2011; Sanderson et al., 2022). Given the constraints associated with the absence of a randomized controlled trial (RCT) or the initiation of new RCTs, this approach emerges as a significant alternative strategy. It offers a reliable foundation for establishing evidence of a causal link between exposure and disease risk (Bowden et al., 2015).

This study aims to investigate the potential causal relationship between nine cathepsins (B, E, F, G, H, O, S, L2, Z) and the risk of developing PD. To comprehensively explore the relationship between cathepsins and PD, bidirectional two-sample MR analysis, multivariable MR analysis, and meta-analysis were employed. This research methodology has enhanced the comprehension of the pathogenic mechanisms underlying PD, while concurrently furnishing robust evidence for the formulation of viable screening and preventive strategies for PD.

## 2 Materials and methods

### 2.1 Study design

In this study, bidirectional two-sample MR and multivariable MR analyses were conducted to systematically assess the intrinsic associations between nine cathepsins (B, E, F, G, H, O, S, L2, Z) and the risk of developing PD. To ensure the credibility of the study design, a series of statistical methods were employed to validate the results. Two distinct PD GWAS datasets were selected, with the R9 dataset utilized for initial analyses and a series of sensitivity tests, while the R10 dataset was employed for replication analysis. Additionally, a meta-analysis was performed to strengthen the overall findings. In order to explore the specific mechanism of cathepsin’s influence on PD, we also tried to conduct two sample MR between the screened cathepsin and alpha-synuclein to verify the possibility of cathepsin’s influence on PD through the regulation of alpha-synuclein.

**Figure 1:**
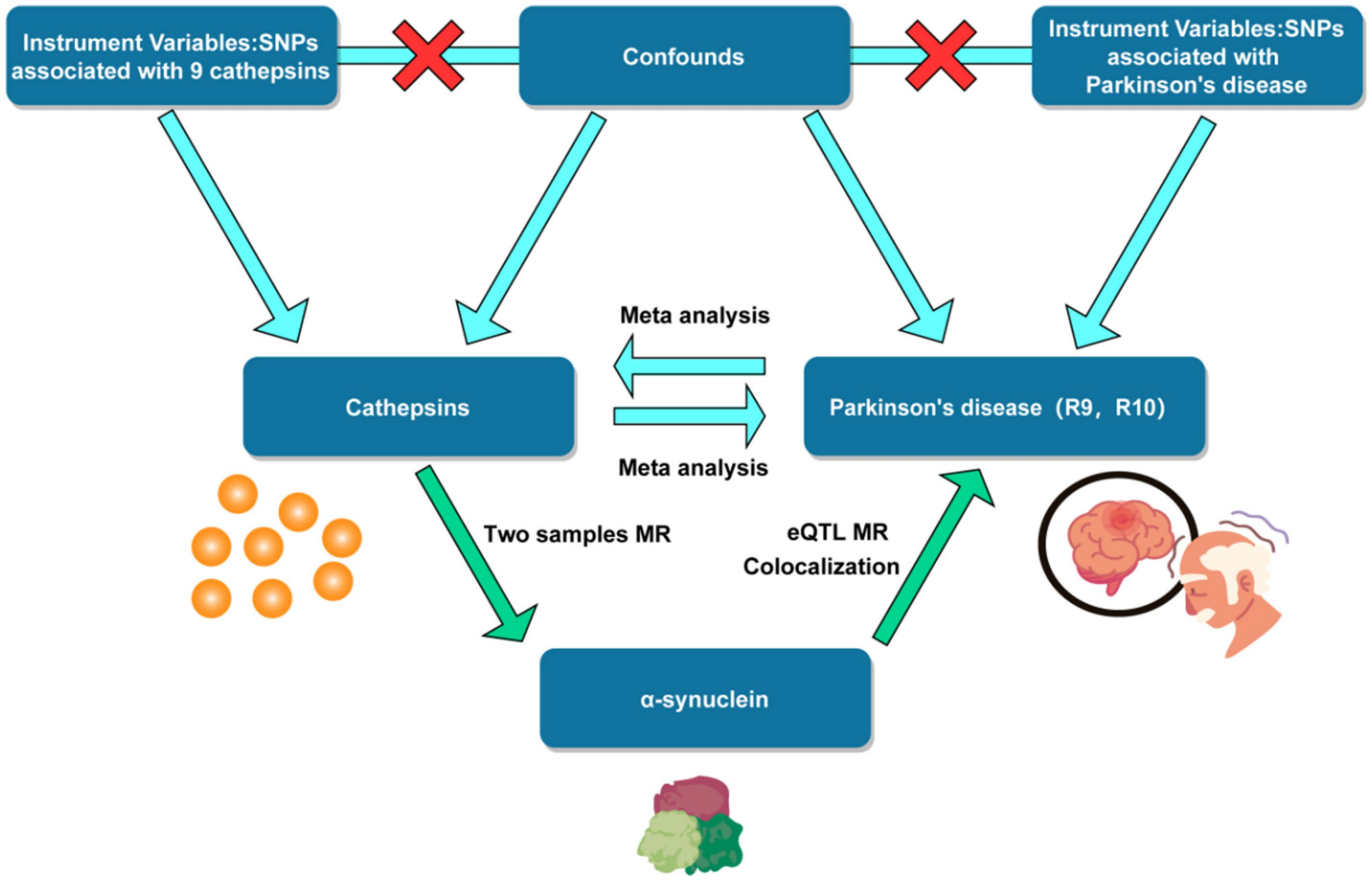
Flow chart of mendelian randomization study design. SNP, single nucleotide polymorphism. eQTL, expression quantitative trait loci.

This MR study encompassed the testing of three hypotheses: Firstly, the genetic IVs are closely correlated with the exposure of interest. Secondly, the genetic IVs are unrelated to the outcome, independent of any known or unknown confounding factors. Furthermore, the impact of IVs on the outcome is solely mediated through the exposure of interest (Boef et al., 2015). A summary of the study design can be found in Figure 1.

### 2.2 Data source

The data on cathepsin and alpha-synuclein were derived from the genomic atlas study of the human plasma proteome conducted by Sun et al. (2018), representing one of the most comprehensive plasma protein GWAS dataset currently available. These findings were sourced from the Integrative Epidemiology Unit (IEU) Open GWAS project, accessible online at https://gwas.mrcieu.ac.uk/ This resource provides a pivotal basis for exploring genetic associations across a wide array of plasma proteins, thereby facilitating a deeper understanding of their roles in human biology and disease. PD data were sourced from the FinnGen database,^1^ with separate collections of PD GWAS data at R9 and R10 levels. All participants included in the GWAS were of European descent (Table 1).

**Table 1:** Details of GWAS summary data.

GWAS	Database	Id/phenocode	Sample size	Population
Cathepsin B	IEU	prot-a-718	3,301	European
Cathepsin E	IEU	prot-a-720	3,301	European
Cathepsin F	IEU	prot-a-722	3,301	European
Cathepsin G	IEU	prot-a-723	3,301	European
Cathepsin H	IEU	prot-a-725	3,301	European
Cathepsin O	IEU	prot-a-726	3,301	European
Cathepsin S	IEU	prot-a-727	3,301	European
Cathepsin L2	IEU	prot-a-728	3,301	European
Cathepsin Z	IEU	prot-a-729	3,301	European
Alpha-synuclein	IEU	prot-a-2789	3,301	European
Parkinson’s disease_R9	FinnGen	G6_PARKINSON	377,277	European
Parkinson’s disease_R10	FinnGen	G6_PARKINSON	412,181	European

GWAS, genome-wide association study; IEU, integrative epidemiology unit.

### 2.3 Selection of instrumental variables

For Cathepsins, single-nucleotide polymorphisms (SNPs) strongly associated with GWAS data are typically limited. Applying a threshold of *p* < 5 × 10^−8^ may result in an insufficient number of IVs for subsequent analysis. Therefore, more relaxed *p*-value criteria are generally employed in these studies. Based on previous research (Chai et al., 2022; Li et al., 2023), a threshold of *p* < 1 × 10^−5^ was used to select IVs. To ensure the independence of the selected IVs, linkage disequilibrium (LD) was assessed with PLINK, using an LD r^2^ < 0.01 within a 10,000 kb window. Furthermore, the strength of each selected SNP was evaluated by calculating the F-statistic using the formula (Pierce et al., 2011). F = [(N - k - 1) / k] × [R^2^ / (1 - R^2^)], where R^2^ indicates the proportion of exposure variance explained by the IVs, k denotes the number of IVs, and N represents the sample size. An F-statistic of 10 or higher provides strong evidence against the presence of weak instrument bias. Conversely, IVs with an F-statistic below 10 are considered weak and are recommended for exclusion (Davies et al., 2018).

### 2.4 Mendelian randomization analysis

Using the TwoSample MR package (version 0.5.6) in R 4.3.2, we investigated the causal relationship between each cathepsin and PD. The inverse variance-weighted method (IVW) was employed as the primary approach for evaluating causal association effects (Burgess et al., 2013). IVW is a meta-summary technique in MR that analyzes the impact of multiple SNPs on various loci when assessing causality. Furthermore, we employed the weighted median method (WME) (Bowden et al., 2016) and MR-Egger regression (Hartwig and Davies, 2016) to assess the reliability and stability of the results. When estimates of causal effects from these three distinct MR models were concordant, we concluded that the causal relationship between cathepsin and PD is reliable.

### 2.5 Heterogeneity and sensitivity tests

Cochran’s Q-test for IVW and MR-Egger was used to detect potential violations of the assumption by the heterogeneity of the association between individual IVs (Cohen et al., 2015). The included IVs will be considered not heterogeneous when *p* > 0.05. The default fixed-effects model was employed if no substantial heterogeneity (*p* > 0.05) was observed; otherwise, the random-effects model was utilized. MR-Egger was applied to estimate horizontal pleiotropy according to its intercept, ensuring that genetic variation was independently associated with exposure and outcome. When *p* > 0.05, it will be considered that there is less likely genetic pleiotropy in the causal analysis. The MR-PRESSO outlier test was used to correct for horizontal pleiotropy by removing or down-weighting the outliers when the horizontal pleiotropy was significant (*p* < 0.05) (Hemani et al., 2018). Furthermore, this study used the leave-one-out method to assess the likelihood of associations observed by individual SNP drivers (Burgess and Thompson, 2017).

## 3 Results

### 3.1 Causal effects of cathepsin on PD

After selecting SNPs with a *p*-value <1 × 10^−5^ and removing SNPs with linkage disequilibrium, we extracted a total of 131 SNPs from GWAS data (Supplementary Table S1). The *F*-value was calculated for each SNP, and the minimum *F*-value among all SNPs was 20.798, no SNP was considered a weak IV and thus excluded (Supplementary Table S2).

**Figure 2:**
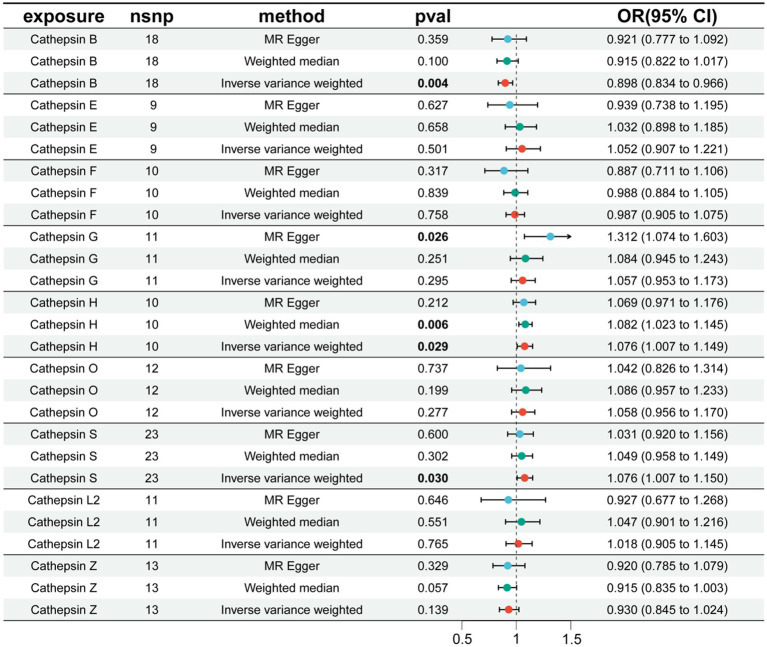
The effect of cathepsin on PD. nSNP, number of single nucleotide polymorphism; OR, odds ratios; CI, confidence interval.

We conducted MR analysis on each cathepsin and PD. The results revealed that CTSB has an inhibitory effect on the occurrence of PD (OR = 0.898, 95% CI: 0.834–0.966, *p* = 0.004), whereas cathepsin H (CTSH) (OR = 1.076, 95% CI: 1.007–1.149, *p* = 0.029) and cathepsin S (CTSS) (OR = 1.076, 95% CI: 1.007–1.150, *p* = 0.030) increase the risk of PD (Figure 2). The robustness of the results was verified by heterogeneity and sensitivity tests (*p* > 0.05) (Supplementary Table S3).

### 3.2 Validation and meta-analysis

**Figure 3:**
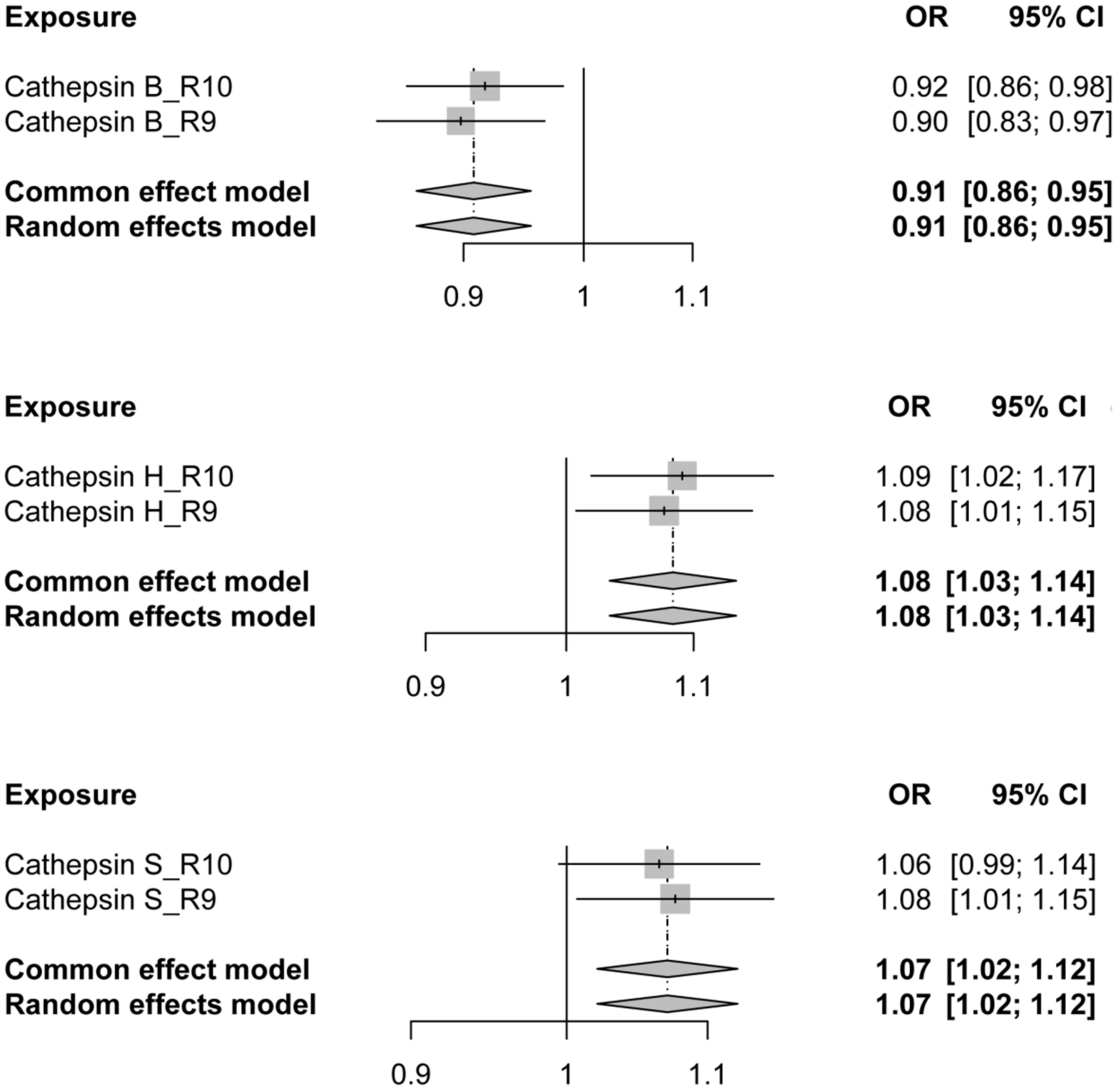
The forest plot depicting the meta-analysis of Mendelian Randomization analysis results. OR, odds ratios; CI, confidence interval.

To further validate the reliability of our conclusions, we reanalyzed the PD GWAS data at R10 level from the FinnGen database, employing it as the outcome. Subsequently, an MR analysis was conducted, and the results of the two analyses were subjected to meta-analysis. The meta-analysis outcomes consistently indicate a protective effect of CTSB against the occurrence of PD, while CTSH and CTSS are associated with an increased risk of PD, aligning with the findings of our previous MR analysis (Figure 3).

### 3.3 Multivariable and reverse MR analysis

To explore the distinct impacts of each cathepsin on PD, a multivariable MR analysis was conducted. Interestingly, we observed that the causal effects of each metabolite were consistent in direction and magnitude with the unadjusted results using the IVW method (Table 2).

**Table 2:** Results of multivariate Mendelian randomization.

Exposure	Method	nSNP	pval	OR	95% CI
Cathepsin B	MVMR	1	0.004	0.896	0.830–0.966
Cathepsin E	MVMR	0	0.447	1.033	0.950–1.123
Cathepsin F	MVMR	3	0.600	1.026	0.932–1.130
Cathepsin G	MVMR	0	0.424	1.039	0.947–1.139
Cathepsin H	MVMR	2	0.004	1.085	1.027–1.146
Cathepsin O	MVMR	0	0.627	1.033	0.907–1.176
Cathepsin S	MVMR	1	0.013	1.092	1.019–1.171
Cathepsin L2	MVMR	1	0.125	1.094	0.975–1.228
Cathepsin Z	MVMR	2	0.074	0.919	0.839–1.008

MVMR, multivariate Mendelian randomization. nSNP, number of single nucleotide polymorphism; OR, odds ratios; CI, confidence interval.

**Figure 4:**
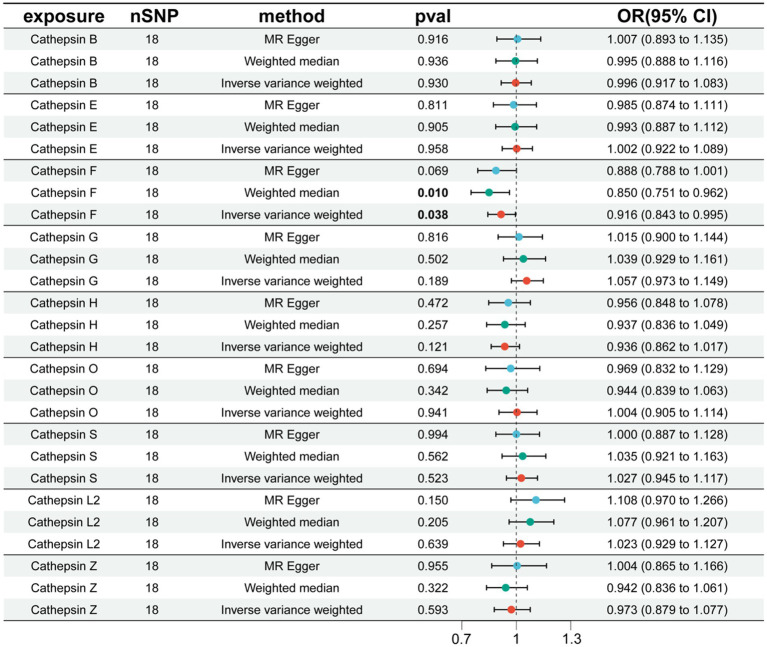
The effect of PD on cathepsin. nSNP, number of single nucleotide polymorphism; OR, odds ratios; CI, confidence interval.

**Figure 5:**
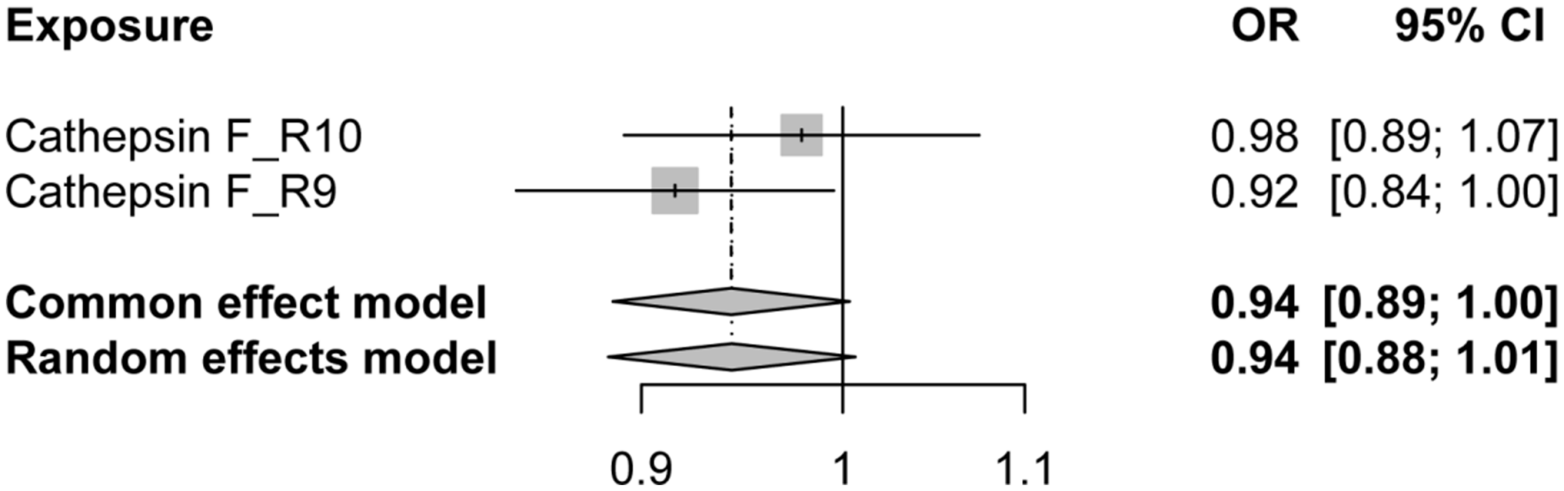
The forest plot of reverse MR meta-analysis results. OR, odds ratios; CI, confidence interval.

Additionally, to examine whether PD has a reverse regulatory effect on cathepsins, a reverse MR analysis was performed. The results suggest a potential decrease in Cathepsin F (CTSF) levels attributable to PD (OR = 0.916, 95% CI: 0.834–0.995, *p* = 0.038) (Figure 4). To ensure the robustness and reproducibility of this finding, analogous to prior similar studies, we utilized R10 level PD data as the exposure variable and conducted replication analyses and meta-analyses for each cathepsin. The aggregated results, however, did not exhibit statistical significance (*p* = 0.065). Consequently, we infer that the inverse regulatory effect of PD on CTSF levels may not be reliable (Figure 5).

### 3.4 Exploration of regulation mechanism

In order to explore the specific mechanism, we executed a two-sample MR using significant cathepsins as the exposure and alpha-synuclein as the outcome. Results displayed that CTSB could reduce the levels of alpha-synuclein (OR = 0.909, 95%CI: 0.841–0.983, *p* = 0.017). No statistical significance was observed in the other two types of cathepsins (Table 3).

**Table 3:** The effect of significant cathepsins on alpha-synuclein.

Exposure	Outcome	Method	nSNP	pval	OR	95% CI
Cathepsin B	Alpha-synuclein	Inverse variance weighted	19	0.032	0.913	0.840–0.992
Cathepsin B	Alpha-synuclein	MR Egger	19	0.609	0.948	0.774–1.160
Cathepsin B	Alpha-synuclein	Weighted median	19	0.207	0.929	0.828–1.042
Cathepsin H	Alpha-synuclein	Method	11	0.109	0.945	0.881–1.013
Cathepsin H	Alpha-synuclein	Inverse variance weighted	11	0.890	0.994	0.908–1.087
Cathepsin H	Alpha-synuclein	MR Egger	11	0.350	0.971	0.912–1.033
Cathepsin S	Alpha-synuclein	Inverse variance weighted	24	0.575	0.977	0.900–1.060
Cathepsin S	Alpha-synuclein	MR Egger	24	0.391	1.062	0.928–1.214
Cathepsin S	Alpha-synuclein	Weighted median	24	0.873	0.992	0.903–1.090

nSNP, number of single nucleotide polymorphism; OR, odds ratios; CI, confidence interval.

**Figure 6:**
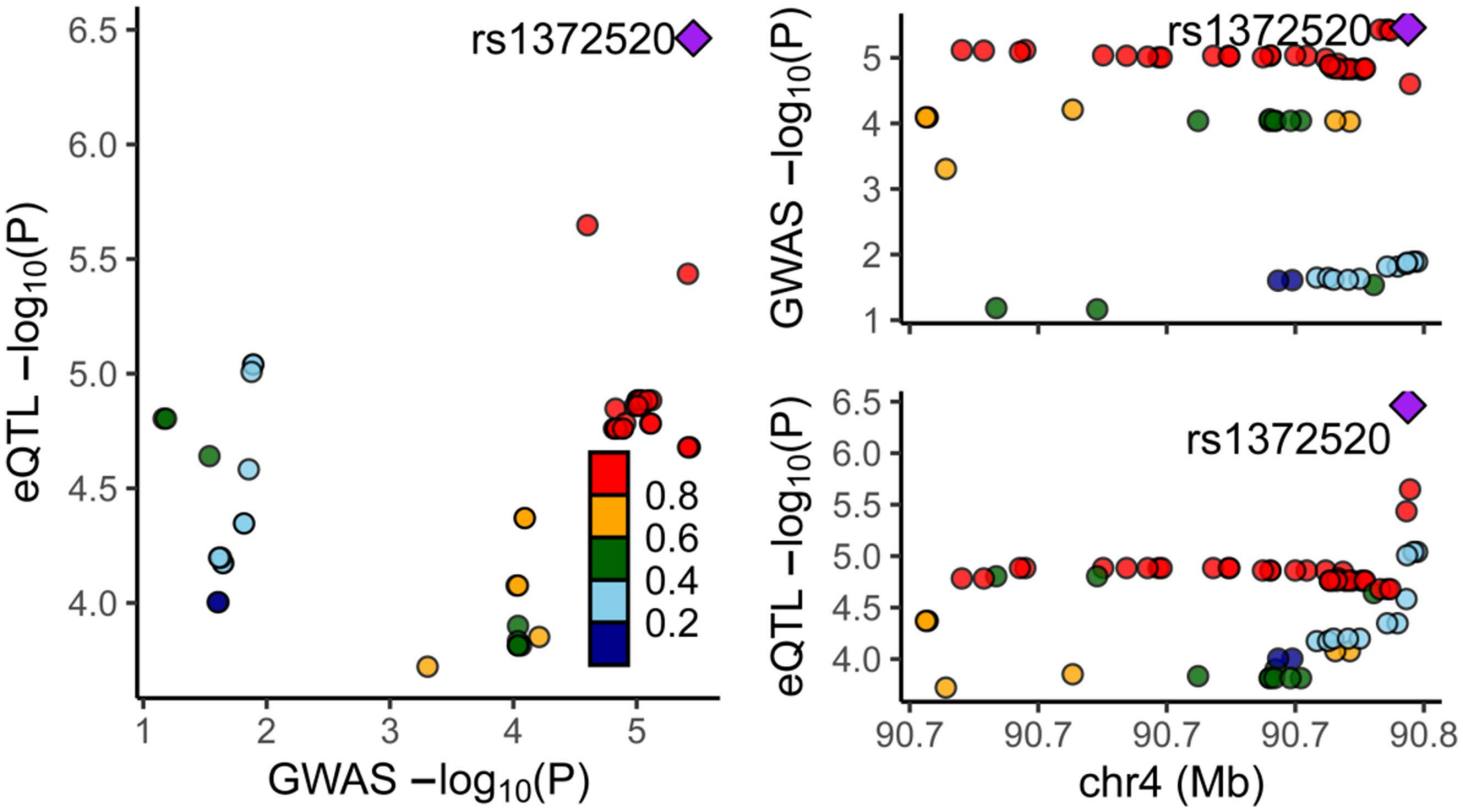
Results of colocalization analysis between the SNCA Gene and PD. Each dot represents a gene, with color indicating the magnitude of the *p*-value. The square denotes the most significant regulatory gene, rs1372520, which concurrently modulates the expression of both SNCA and PD.

To assess the association between alpha-synuclein and PD, a thorough search for genes closely linked with alpha-synuclein was undertaken in the National Center for Biotechnology Information database. The strongest relationship was found between the SNCA gene and PD, prompting our use of SNCA-related eQTL data for a two-sample MR, and a colocalization analysis with PD. The MR results consistently showed that the expression of the SNCA gene could increase the onset of PD (OR = 3.344, 95%CI: 2.465–4.537, *p* = 8.540E-15) (Supplementary Table S4). The colocalization analysis suggested that alpha-synuclein and PD are driven by the same causal variant within a specific region, demonstrating an inherent genetic linkage (PP.H4.abf =0.972) (Figure 6; Supplementary Table S5). Thus, it can be inferred that by reducing the level of alpha-synuclein, CTSB could potentially attenuate the risk of PD onset. This finding aligns with our previous forward MR analysis results.

## 4 Discussion

This study provided compelling evidence for the distinct roles of CTSB, CTSH, and CTSS in the pathogenesis of PD. Through MR analysis, we established a nuanced understanding of how these proteases might influence PD risk, suggesting a protective role for CTSB and identifying cathepsins H and S as potential risk factors. These findings offer a significant advancement in our comprehension of PD’s etiology.

The findings of our study suggest that enhancing CTSB activity could mitigate the progression of PD by promoting the degradation of alpha-synuclein. This observation aligns well with the cellular experiments conducted by Jace Jones-Tabah et al. (2023), which demonstrated that reduced expression of the CTSB gene impairs the degradation of preformed alpha-synuclein fibrils in cell lines. This correlation underscores the potential of CTSB activity modulation as a therapeutic strategy for PD.

The core protein implicated in PD pathogenesis is alpha-synuclein, and strategies aimed at reducing its production, diminishing aggregation, and promoting its degradation are pivotal for therapeutic design. The noteworthy observation that CTSB may mitigate the risk of PD by reducing alpha-synuclein levels underscores the significance of alpha-synuclein clearance as a crucial therapeutic target (Bennett, 2005). On one hand, the most prevalent pathogenic mutations associated with autosomal dominant Parkinson’s disease involved the leucine-rich repeat kinase 2 (LRRK2) gene, with the most prominent being the G2019S mutation. LRRK2 mutations enhance the incorporation of alpha-synuclein into neuronal inclusions. The formation of alpha-synuclein fibril-induced intracellular aggregates necessitates lysosomal function, which, in turn, relies on CTSB activity (Tsujimura et al., 2015). The reduced activity of CTSB induced by the LRRK2 G2019S mutation potentially contributes to the aberrant accumulation of alpha-synuclein within neurons (Hu et al., 2018). Hence, augmentation in both the activity and quantity of CTSB may lead to a reduction in alpha-synuclein aggregation, thereby offering therapeutic potential for PD (Volpicelli-Daley et al., 2016). On the other hand, alpha-synuclein is primarily degraded within lysosomes, wherein CTSB plays a critical role. Studies by McGlinchey and Lee (2015) on purified mouse brain and lysosomal extracts, as well as human tissue proteases, suggest that CTSB directly participates in the initial peptide cleavage of alpha-synuclein during lysosomal degradation and plays a crucial role in truncating the C-terminal of alpha-synuclein (McGlinchey et al., 2019).

Conversely, the association of cathepsins H and S with increased PD risk underscores the complexity of cathepsins’ roles in neurodegeneration. While these enzymes are essential for protein turnover and immune regulation, their dysregulation may contribute to neuronal damage and inflammation, exacerbating PD pathology (Stoka et al., 2016). This dual role of cathepsins highlights the importance of context-specific functions and the delicate balance between their protective and harmful effects in the brain.

The upregulation of CTSH could potentially contribute to neurodegeneration in PD. It is well-established that the accumulation of alpha-synuclein in Lewy bodies is a pathological hallmark of PD, and the abnormal aggregation of alpha-Syn activates microglia, leading to neuroinflammation, which is neurotoxic (Sanchez-Guajardo et al., 2015). PD is a neurodegenerative disease, and lysosomal dysfunction, attributed to the accumulation and spread of neurotoxic protein oligomers, including alpha-synuclein, is common in most neurodegenerative diseases, with CTSH playing a significant role (Boland et al., 2018; Wang et al., 2018). The abnormal accumulation of proteins such as amyloid-beta and alpha-synuclein activates microglia, inducing the activation and release of CTSH and pro-inflammatory cytokines, including interleukin-1β and tumor necrosis factor-alpha, further exacerbating this self-propagating neurotoxicity, leading to neurodegeneration in PD. This aligns with and extends prior research implicating the lysosomal pathway and autophagy processes in neurodegeneration (Senkevich and Gan-Or, 2020).

Studies have revealed the significant involvement of CTSS in diseases such as Alzheimer’s disease, acute brain injury, stroke, diabetes, liver cancer, and inflammation. Through proteomic analysis of cerebrospinal fluid and urine from PD patients, researchers have identified several potential biomarkers associated with PD, among which CTSS levels were significantly increased in the cerebrospinal fluid of carriers of the LRRK2 G2019S mutation. These findings suggest a role for CTSS in the pathogenesis of PD, potentially mediated through enhanced inflammatory responses (Stojkovska et al., 2015; Caggiu et al., 2019). Zhang et al. (2022) explored platelet biomarkers for PD diagnosis by isolating PLTs from whole blood samples of PD patients and healthy controls for RNA sequencing. They identified 2,221 genes with differential transcriptional levels, among which CTSS was associated with PD among the top 12 most relevant genes.

However, our study has limitations that warrant consideration. The reverse MR analysis did not yield significant results, suggesting that the causal direction between cathepsin levels and PD might be complex and influenced by factors not accounted for in our study. Additionally, while MR provides robust evidence for causality under certain assumptions, it is essential to acknowledge the potential for pleiotropy and confounding factors that could affect the interpretation of our results.

Future research should focus on elucidating the mechanistic pathways through which cathepsins influence PD risk and progression. Clinical investigations, including longitudinal studies and randomized controlled trials, are crucial to confirm our findings and explore the therapeutic potential of modulating cathepsin activity. Additionally, investigating the interaction between cathepsins and other key players in PD pathology, such as LRRK2 and glucocerebrosidase, could provide a more comprehensive understanding of the disease mechanism.

## 5 Conclusion

In summary, this study establishes a causal relationship between cathepsins and PD. Our forward MR analysis indicates that more CTSB associated with less PD risk, while more CTSH and CTSS may associated with increased PD risk. Additionally, we observed that CTSB may mitigate PD development by reducing alpha-synuclein levels. Nevertheless, further clinical investigations are warranted to elucidate the precise association between Cathepsins and PD, uncover the underlying mechanisms, and advance the prevention and treatment of PD.

## Data availability statement

Publicly available datasets were analyzed in this study. This data can be found here: The data and material that support the findings of this study are available in the IEU Open GWAS (https://gwas.mrcieu.ac.uk/datasets/) and FinnGen consortium (https://www.finngen.fi/fi).

## Ethics statement

In accordance with local legislative and institutional requirements, this research involving human participants did not necessitate ethical approval. Per national legislation and institutional directives, the study was exempt from the requirement to obtain written informed consent from the subjects or their legal guardians/close relatives. The studies were conducted in accordance with the local legislation and institutional requirements. Written informed consent for participation was not required from the participants or the participants' legal guardians/next of kin in accordance with the national legislation and institutional requirements.

## Author contributions

LL: Data curation, Methodology, Software, Visualization, Writing – original draft, Writing – review & editing. ZW: Data curation, Software, Visualization, Writing – original draft. HL: Conceptualization, Investigation, Writing – original draft. YH: Supervision, Validation, Writing – original draft, Writing – review & editing.

## Footnotes

^1^ https://www.finngen.fi/fi

## References

[ref1] Bennett M. C. (2005). The role of α-synuclein in neurodegenerative diseases. Pharmacol. Ther. 105, 311–331. doi: 10.1016/j.pharmthera.2004.10.010 15737408

[ref2] Blauwendraat C. Reed X. Krohn L. Heilbron K. Bandres-Ciga S. Tan M.. (2020). Genetic modifiers of risk and age at onset in gba associated parkinson's disease and lewy body dementia. Brain 143, 234–248. doi: 10.1093/brain/awz350 31755958 PMC6935749

[ref3] Bloem B. R. Okun M. S. Klein C. (2021). Parkinson's disease. Lancet 397, 2284–2303. doi: 10.1016/S0140-6736(21)00218-X 33848468

[ref4] Boef A. G. Dekkers O. M. le Cessie S. (2015). Mendelian randomization studies: a review of the approaches used and the quality of reporting. Int. J. Epidemiol. 44, 496–511. doi: 10.1093/ije/dyv071, PMID: 25953784

[ref5] Boland B. Yu W. H. Corti O. Mollereau B. Henriques A. Bezard E.. (2018). Promoting the clearance of neurotoxic proteins in neurodegenerative disorders of ageing. Nat. Rev. Drug Discov. 17, 660–688. doi: 10.1038/nrd.2018.109, PMID: 30116051 PMC6456907

[ref6] Bowden J. Davey S. G. Burgess S. (2015). Mendelian randomization with invalid instruments: effect estimation and bias detection through egger regression. Int. J. Epidemiol. 44, 512–525. doi: 10.1093/ije/dyv080, PMID: 26050253 PMC4469799

[ref7] Bowden J. Davey S. G. Haycock P. C. Burgess S. (2016). Consistent estimation in mendelian randomization with some invalid instruments using a weighted median estimator. Genet. Epidemiol. 40, 304–314. doi: 10.1002/gepi.21965, PMID: 27061298 PMC4849733

[ref8] Burgess S. Butterworth A. Thompson S. G. (2013). Mendelian randomization analysis with multiple genetic variants using summarized data. Genet. Epidemiol. 37, 658–665. doi: 10.1002/gepi.21758, PMID: 24114802 PMC4377079

[ref9] Burgess S. Thompson S. G. (2017). Interpreting findings from mendelian randomization using the mr-egger method. Eur. J. Epidemiol. 32, 377–389. doi: 10.1007/s10654-017-0255-x, PMID: 28527048 PMC5506233

[ref10] Caggiu E. Arru G. Hosseini S. Niegowska M. Sechi G. Zarbo I. R.. (2019). Inflammation, infectious triggers, and parkinson's disease. Front. Neurol. 10:122. doi: 10.3389/fneur.2019.00122, PMID: 30837941 PMC6389614

[ref11] Chai T. Tian M. Yang X. Qiu Z. Lin X. Chen L. (2022). Association of circulating cathepsin b levels with blood pressure and aortic dilation. Front. Cardiovasc. Med. 9:762468. doi: 10.3389/fcvm.2022.762468, PMID: 35425820 PMC9001941

[ref12] Cohen J. F. Chalumeau M. Cohen R. Korevaar D. A. Khoshnood B. Bossuyt P. M. (2015). Cochran's q test was useful to assess heterogeneity in likelihood ratios in studies of diagnostic accuracy. J. Clin. Epidemiol. 68, 299–306. doi: 10.1016/j.jclinepi.2014.09.005, PMID: 25441698

[ref13] Davies N. M. Holmes M. V. Davey S. G. (2018). Reading mendelian randomisation studies: a guide, glossary, and checklist for clinicians. BMJ 362:k601. doi: 10.1136/bmj.k601, PMID: 30002074 PMC6041728

[ref14] Dehay B. P. Bourdenx M. M. Gorry P. M. Przedborski S. P. Vila M. M. Hunot S. P.. (2015). Targeting α-synuclein for treatment of parkinson's disease: mechanistic and therapeutic considerations. Lancet Neurol. 14, 855–866. doi: 10.1016/S1474-4422(15)00006-X, PMID: 26050140 PMC5217462

[ref15] Hartwig F. P. Davies N. M. (2016). Why internal weights should be avoided (not only) in mr-egger regression. Int. J. Epidemiol. 45, 1676–1678. doi: 10.1093/ije/dyw240, PMID: 27649799

[ref16] Hemani G. Bowden J. Davey S. G. (2018). Evaluating the potential role of pleiotropy in mendelian randomization studies. Hum. Mol. Genet. 27, R195–R208. doi: 10.1093/hmg/ddy163 29771313 PMC6061876

[ref17] Hu D. Niu J. Xiong J. Nie S. Zeng F. Zhang Z. (2018). Lrrk2 g2019s mutation inhibits degradation of α-synuclein in an in vitro model of parkinson’s disease. Curr. Med. Sci. 38, 1012–1017. doi: 10.1007/s11596-018-1977-z, PMID: 30536063

[ref18] Jones-Tabah J. He K. Senkevich K. Karpilovsky N. Deyab G. Cousineau Y.. (2023). The parkinson's disease risk gene cathepsin b promotes fibrillar alpha-synuclein clearance, lysosomal function and glucocerebrosidase activity in dopaminergic neurons. *Biorxiv*. [Epub ahead of preprint]. doi: 10.1101/2023.11.11.566693, PMID: 38014143 PMC10680785

[ref19] Khaket T. P. Kwon T. K. Kang S. C. (2019). Cathepsins: potent regulators in carcinogenesis. Pharmacol. Ther. 198, 1–19. doi: 10.1016/j.pharmthera.2019.02.003 30763594

[ref20] Li J. Tang M. Gao X. Tian S. Liu W. (2023). Mendelian randomization analyses explore the relationship between cathepsins and lung cancer. Commun. Biol. 6:1019. doi: 10.1038/s42003-023-05408-7, PMID: 37805623 PMC10560205

[ref21] McGlinchey R. P. Lacy S. M. Huffer K. E. Tayebi N. Sidransky E. Lee J. C. (2019). C-terminal α-synuclein truncations are linked to cysteine cathepsin activity in parkinson’s disease. J. Biol. Chem. 294, 9973–9984. doi: 10.1074/jbc.RA119.008930, PMID: 31092553 PMC6597809

[ref22] McGlinchey R. P. Lee J. C. (2015). Cysteine cathepsins are essential in lysosomal degradation of alpha-synuclein. Proc. Natl. Acad. Sci. USA 112, 9322–9327. doi: 10.1073/pnas.1500937112, PMID: 26170293 PMC4522768

[ref23] Milanowski L. M. Hou X. Bredenberg J. M. Fiesel F. C. Cocker L. T. Soto-Beasley A. I.. (2022). Cathepsin b p.gly284val variant in parkinson's disease pathogenesis. Int. J. Mol. Sci. 23:7086. doi: 10.3390/ijms23137086, PMID: 35806091 PMC9266886

[ref24] Moors T. Paciotti S. Chiasserini D. Calabresi P. Parnetti L. Beccari T.. (2016). Lysosomal dysfunction and alpha-synuclein aggregation in parkinson's disease: diagnostic links. Mov. Disord. 31, 791–801. doi: 10.1002/mds.26562, PMID: 26923732

[ref25] Pierce B. L. Ahsan H. Vanderweele T. J. (2011). Power and instrument strength requirements for mendelian randomization studies using multiple genetic variants. Int. J. Epidemiol. 40, 740–752. doi: 10.1093/ije/dyq151, PMID: 20813862 PMC3147064

[ref26] Sanchez-Guajardo V. Tentillier N. Romero-Ramos M. (2015). The relation between α-synuclein and microglia in parkinson’s disease: recent developments. Neuroscience 302, 47–58. doi: 10.1016/j.neuroscience.2015.02.008, PMID: 25684748

[ref27] Sanderson E. Glymour M. M. Holmes M. V. Kang H. Morrison J. Munafò M. R.. (2022). Mendelian randomization. Nat. Rev. Methods Primers 2:6. doi: 10.1038/s43586-021-00092-5, PMID: 37325194 PMC7614635

[ref28] Senkevich K. Gan-Or Z. (2020). Autophagy lysosomal pathway dysfunction in parkinson's disease; evidence from human genetics. Parkinsonism Relat. Disord. 73, 60–71. doi: 10.1016/j.parkreldis.2019.11.015 31761667

[ref29] Smith G. D. Ebrahim S. (2003). 'mendelian randomization': can genetic epidemiology contribute to understanding environmental determinants of disease? Int. J. Epidemiol. 32, 1–22. doi: 10.1093/ije/dyg070 12689998

[ref30] Stojkovska I. Wagner B. M. Morrison B. E. (2015). Parkinson’s disease and enhanced inflammatory response. Exp. Biol. Med. 240, 1387–1395. doi: 10.1177/1535370215576313, PMID: 25769314 PMC4935292

[ref31] Stoka V. Turk V. Turk B. (2016). Lysosomal cathepsins and their regulation in aging and neurodegeneration. Ageing Res. Rev. 32, 22–37. doi: 10.1016/j.arr.2016.04.010 27125852

[ref001] Sun B. B. Maranville J. C. Peters J. E. Stacey D. Staley J. R. Blackshaw J.. (2018). Genomic atlas of the human plasma proteome. Nature. 558, 73–79. doi: 10.1038/s41586-018-0175-2, PMID: 29875488 PMC6697541

[ref32] Tsujimura A. Taguchi K. Watanabe Y. Tatebe H. Tokuda T. Mizuno T.. (2015). Lysosomal enzyme cathepsin b enhances the aggregate forming activity of exogenous α-synuclein fibrils. Neurobiol. Dis. 73, 244–253. doi: 10.1016/j.nbd.2014.10.011 25466281

[ref33] Vizovišek M. Fonović M. Turk B. (2019). Cysteine cathepsins in extracellular matrix remodeling: extracellular matrix degradation and beyond. Matrix Biol. 75-76, 141–159. doi: 10.1016/j.matbio.2018.01.024, PMID: 29409929

[ref34] Volpicelli-Daley L. A. Abdelmotilib H. Liu Z. Stoyka L. Daher J. P. Milnerwood A. J.. (2016). G2019s-lrrk2 expression augments alpha-synuclein sequestration into inclusions in neurons. J. Neurosci. 36, 7415–7427. doi: 10.1523/JNEUROSCI.3642-15.2016, PMID: 27413152 PMC4945663

[ref35] Wang H. Inoue A. Lei Y. Wu H. Hong L. Cheng X. W. (2023). Cathepsins in the extracellular space: focusing on non-lysosomal proteolytic functions with clinical implications. Cell. Signal. 103:110531. doi: 10.1016/j.cellsig.2022.110531, PMID: 36417977

[ref36] Wang C. Telpoukhovskaia M. A. Bahr B. A. Chen X. Gan L. (2018). Endo-lysosomal dysfunction: a converging mechanism in neurodegenerative diseases. Curr. Opin. Neurobiol. 48, 52–58. doi: 10.1016/j.conb.2017.09.005, PMID: 29028540

[ref37] Zhang L. Shao Y. Tang C. Liu Z. Tang D. Hu C.. (2022). Identification of novel biomarkers in platelets for diagnosing parkinson’s disease. Eur. Neurol. 85, 122–131. doi: 10.1159/000520102, PMID: 34875658

